# Child and adolescent internalizing and externalizing problems 12 months postburn: the potential role of preburn functioning, parental posttraumatic stress, and informant bias

**DOI:** 10.1007/s00787-015-0788-z

**Published:** 2015-11-25

**Authors:** Marthe R. Egberts, Rens van de Schoot, Anita Boekelaar, Hannelore Hendrickx, Rinie Geenen, Nancy E. E. Van Loey

**Affiliations:** Association of Dutch Burn Centres, P. O. Box 1015, 1940 EA Beverwijk, The Netherlands; Department of Clinical and Health Psychology, Utrecht University, Utrecht, The Netherlands; Department of Methodology and Statistics, Faculty of Social Sciences, Utrecht University, Utrecht, The Netherlands; Optentia Research Program, Faculty of Humanities, North-West University, Vanderbijlpark, South Africa; Burn Centre Red Cross Hospital, Beverwijk, The Netherlands; Burn Centre University Hospital Gent, Ghent, Belgium

**Keywords:** Burns, Behavioral problems, Children, Preburn functioning, Parental posttraumatic stress, Cross-informant agreement

## Abstract

Adjustment after pediatric burn injury may be a challenge for children as well as their parents. This prospective study examined associations of internalizing and externalizing problems in children and adolescents 12 months postburn with preburn functioning, and parental acute and chronic posttraumatic stress symptoms (PTSS) from different perspectives. Child, mother, and father reports of 90 children (9–18 years), collected within the first month and 12 months postburn, were analyzed. Results indicated that overall, child and parental appraisals of pre- and postburn behavioral problems were not significantly different from reference data. Rates of (sub)clinical postburn behavioral problems ranged from 6 to 17 %, depending on the informant. Pre- and postburn behavioral problems were significantly related, but only from the parents’ perspective. Path models showed an association between parental PTSS 12 months postburn and parental reports of child internalizing problems, as well as a significant indirect relationship from parental acute stress symptoms via PTSS 12 months postburn. Notably, no associations between parental PTSS and child reports of postburn behavioral problems were found. In conclusion, parental observations of child externalizing problems appear to be influenced by their perspectives on the child’s preburn functioning, while parental observations of internalizing problems are also related to long-term parental PTSS. However, these factors seem of no great value in predicting behavioral problems from the child’s perspective, suggesting substantial informant deviations. To optimize adjustment, clinical burn practice is recommended to adopt a family perspective including parent perception of preburn functioning and parental PTSS in assessment and intervention.

## Introduction

Adjustment after pediatric burn injury may be a challenge for children and adolescents, as well as their parents. Burn events are often traumatic in nature, the wounds are extremely painful, and deep dermal burns inevitably cause disfiguring scars. Children need to adjust to their changed physical appearance and may feel dissatisfaction with appearance [1]. At the same time, they may have to cope with reactions in their social environment and possible stigmatizing behaviors [2]. As a consequence, children could develop internalizing (i.e., symptoms of depression and anxiety) and externalizing (i.e., symptoms of anger and aggression) problems. Adjustment might be especially difficult in (early) adolescence, a period in which more emphasis is placed on outer appearance and social acceptance. Although internalizing and externalizing problems after burn events in children have been a regular topic of previous research, to date, prospective long-term studies for school-aged children and adolescents are lacking.

Earlier cross-sectional studies in school-aged children and adolescents with burns have reported elevated levels of internalizing problems during hospitalization and shortly after this phase [3, 4], as well as elevated levels of externalizing problems [5]. The few cross-sectional studies on long-term behavioral problems (i.e., at least 1 year after the burn event), have examined children from a wide range of ages at burn injury. These studies generally showed no differences between burn-injured children and the normative population [6–9]. Also, specifically in young children (i.e., 0–4 years), cross-sectional as well as prospective studies indicate that the levels of internalizing and externalizing problems in the long term do not deviate substantially from normative groups [10–12]. Nevertheless, a proportion of children (3–13 %) is indicated to experience clinically significant behavioral problems, but factors that play a role in the development of internalizing and externalizing problems are not well understood.

In the literature on burns, inconsistent findings emerge regarding the role of preburn functioning and burn severity in determining postburn outcomes. Some studies in adults find evidence for a role of burn severity in psychological outcomes [13, 14], while other studies suggest that postburn problems are mainly the consequence of a history of preburn psychopathology [15]. In children, mainly cross-sectional studies on long-term internalizing and externalizing problems are available, from which the role of preburn functioning cannot reliably be derived. To our knowledge, only one study on child postburn behavioral problems included appraisals of preburn behavior [16]. This study examined toddlers with large burns and found that postburn rated internalizing and externalizing problems did not differ from retrospective accounts of preburn behavior. More general, a meta-analysis on risk factors of psychopathology following accidental trauma has pointed to pretrauma psychopathology as one of the most important and consistent predictors [17]. Regarding burn severity, the majority of long-term studies have reported that burn size is not related to total behavioral problems [6, 7, 18]. These studies may suggest that most children are able to overcome the consequence of burns irrespective of the burn size and could suggest a role of the family in the child’s adjustment process.

Trauma research indicates that parental posttraumatic stress symptoms (PTSS) are interrelated with the child’s adjustment [19, 20], both in young and in older children, and across a variety of traumatic events, including burn injuries [10, 21, 22]. It is assumed that parents experiencing PTSS possibly are less available to support their child in the posttraumatic period, which may affect the child’s adjustment. Moreover, parental avoidance symptoms, such as not talking about the trauma and avoidance of trauma-related stimuli, could have a negative impact on the child [23]. As a consequence, children may not be able to confront and resolve their own anxieties, hereby maintaining psychological symptoms [24]. Mothers often experience adverse outcomes after a burn event to their child, particularly PTSS [25, 26]. Studies indicate that fathers may also experience these negative outcomes [25, 27], but research on paternal adjustment after pediatric burn injury is scarce. In addition, as most burn studies have focused on parental PTSS and child outcomes of pre-school children, there is little evidence concerning the interrelatedness of parents’ PTSS and postburn functioning of older children and adolescents.

Another explanation for a relationship between parental PTSS and child adjustment may lie in a possible observational bias as a consequence of posttraumatic stress. Research has indicated that parents’ own responses to a traumatic event appear to influence their assessment of child symptoms [28]. Compared to child self-report, highly distressed parents tend to overestimate PTSS in their child [29]. In burn research, a prospective study on 1-year postburn behavioral problems in young children showed that both mothers’ and fathers’ acute stress symptoms predicted higher levels of child postburn internalizing and externalizing problem behavior [10]. As most studies, including the latter, have used the parent as the only informant, it is not clear whether this reflects informant bias or is due to the mutually influencing parent–child interactions. Studies including child reports may add to this currently insufficiently understood association. Furthermore, parental PTSS may reduce and it is not clear how this change may affect parental behavioral observation of the child’s behavioral problems.

**Fig. 1:**
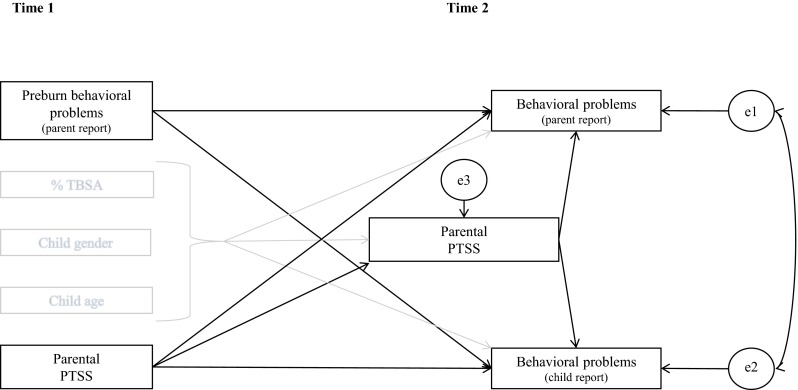
Hypothesized model on the associations between preburn behavioral problems, parental PTSS, and behavioral problems 12 months postburn

The present study will examine the associations between appraisals of preburn functioning, parental PTSS and internalizing and externalizing problems (i.e., behavioral problems) 12 months postburn in children and adolescents (age 9–18 years). Moreover, it is examined whether these associations are different across informants of postburn behavioral problems (children, mothers, and fathers). First, pre- and postburn behavioral problems in our sample will be compared to data from the normative population. Our first hypothesis is that our sample will, on average, not deviate substantially from these reference data [6, 7]. Next, relationships between preburn behavioral problems, parental PTSS, and child postburn behavioral problems will be examined. The hypothesized model is displayed in Fig. 1. Direct relationships of parental PTSS within the first month postburn and PTSS 12 months postburn with child behavioral problems will be studied, as well as an indirect (i.e., mediational) relationship between parental PTSS within the first month postburn and child behavioral problems through parental PTSS 12 months postburn. Our second set of hypotheses concerns these relationships. A significant positive relationship between pre- and postburn behavioral problems is anticipated [17]. It is hypothesized that parents’ own PTSS within the first month postburn will be directly related to observing behavioral problems in the child [10], as well as through parental PTSS 12 months postburn. Direct relationships between parental PTSS 12 months postburn and parental observations of postburn behavioral problems in the child are hypothesized to be significant [28, 29]. However, no significant associations between parental PTSS (at both time points) and children’s self-reported behavioral problems are expected [29].

## Methods

### Procedure

This study is part of a larger prospective study on child (age 8–18 years) and parental adjustment following a pediatric burn event. This cohort has been described in another study, focused on health-related quality of life [30]. The study was approved by two independent ethics committees in the Netherlands and Belgium. From April 2007 to July 2011, data were collected in three Dutch and four Belgian burn centers. The current study used data collected within the first month postburn (T1) and at 12 months postburn (T2). Researchers at the burn centers contacted eligible families during admission and requested to complete the first questionnaires within the first 4 weeks postburn. They explained the study purpose and offered additional written information. Written informed consent was obtained from the mother, father, and child. Families were eligible to participate if the child had been in the hospital for more than 24 h and the total burned surface area (TBSA) was more than or equal to 1 %. Exclusion criteria were insufficient Dutch language proficiency and mental retardation in the child.

A total of 202 families were eligible for the larger study. For the purpose of this paper, we selected children who, at 12 months postburn, had the minimum age (11 years) to fill out the Youth Self-Report (YSR) [31]. A total of 52 children were not in the appropriate age range, leaving 148 children eligible for this study. Of these, 16 families declined to participate in this study, ten families gave their informed consent to participate but dropped out within the first month postburn, 13 children were already discharged before the family could be approached, and 11 families were not invited because their participation was deemed inappropriate (e.g., psychiatric background, multi-problem families, or severely ill family members). In eight families, parents had incomplete data on child preburn functioning and/or their own PTSS. Thus, for the present study, we used data of 90 families that all gave informed consent and from which at least one of the parents provided information on preburn functioning as well as their own PTSS at T1 or T2. We compared the 90 participating families with nonparticipating families and found no significant differences in terms of child gender (*p* = 0.80), age (*p* = 0.63), length of stay in the hospital (*p* = 0.42), percentage TBSA (*p* = 0.35), number of surgeries (*p* = 0.68), and percentage deep burns (*p* = 0.86).

### Participants

Eighty-five mothers and 65 fathers of 90 children were included in final analyses. Demographic and burn characteristics of the children are presented in Table 1. As can be seen, 56 % of the children required at least one surgery.

**Table 1:** Sample demographic and burn characteristics of included children (*n* = 90)

	*M*	SD	Range
Child age (years)	13.9	2.4	9.5–17.8
TBSA (%)	10.0	12.3	1–72
Length of stay in hospital (in days)	19.4	29.7	1–180
Number of surgeries	1.2	2.6	0–16

	*n*	%
Child gender (boys)	65	72
Burn type		
Flame/fire	56	62
Scald	23	26
Contact	4	4
Chemical/electrical	6	7
Other	1	1
Site of accident		
At home (in- or out-side)	53	59
Somewhere else (in- or out-side)	37	41

The mean age of participating mothers was 43.1 years (SD = 5.6, range 28–55 years). The mean age of participating fathers was 45.9 years (SD = 6.2, range 33–64 years). The majority of the parents were employed (75 % of mothers and 91 % of fathers) and had a partner (87 % of mothers and 89 % of fathers).

In between the two studied time points, of the 90 families, 39 children, 30 mothers, and 32 fathers dropped out. However, reports of families with (partially) missing data at T2 were not excluded in further analyses. Comparisons between families that participated at T1 and T2 and families that dropped out revealed no differences in terms of child age (Mothers: *p* = .17, Fathers: *p* = .12), gender (Mothers: *p* = .90, Fathers: *p* = .32), percentage TBSA (Mothers: *p* = .94, Fathers: *p* = .94), preburn behavioral problems (Mothers: *p* = .52, Fathers: *p* = .19), and parental PTSS at T1 (Mothers: *p* = .77, Fathers: *p* = .64).

### Measures

#### Child behavioral problems

Within the first month postburn, both parents completed the Strengths and Difficulties Questionnaire (SDQ) [32], a measure that has been previously used as a brief behavioral screening questionnaire [33]. The SDQ consists of 25 items. Parents were asked to retrospectively report on the functioning of their child prior to the burn event. Because the hospitalization phase is often a demanding period for parents, using a brief questionnaire seemed more convenient than using an extensive and time-consuming questionnaire. Parents were asked to rate behavioral items on a 3-point scale ranging from 0 (not true) to 2 (very true). Items of the SDQ fall within five scales: conduct problems, inattention-hyperactivity, emotional symptoms, peer problems, and prosocial behavior. The total difficulty scores comprise the sum of these scales, excluding the last. For the present study, the Total Difficulties score was used as a predictor variable. Scores higher than 14 on the total scale were referred to as (sub)clinical scores. The reliability and validity of the SDQ are in general satisfactory [34]. In our sample, Cronbach’s alpha for the total SDQ-score was .84 for mother reports and .83 for father reports. SDQ reference data were derived from a British sample consisting of children in between 11 and 15 years old [35], as there were no appropriate Dutch or Belgian reference data available.

At 12 months postburn, both parents completed the Dutch version of the Child Behavior Checklist (CBCL) [36]. Parents rated behavioral items on a 3-point scale ranging from 0 (not true) to 2 (very true or often true). Children filled out the related YSR (YSR) [31], containing the same items as the CBCL. The CBCL and accompanying YSR are extensively validated instruments with adequate reliability and validity [31, 36]. In our sample, Cronbach’s alpha for the internalizing symptoms scale was .84 for mothers, .84 for fathers, and .92 for children (YSR). For the externalizing symptoms scale, coefficients were .90 for mothers, .86 for fathers, and .87 for children. For the CBCL and YSR, *T* scores were used to represent child postburn behavioral problems. Dutch CBCL and YSR reference data were derived from the Dutch manual [37], which were collected as part of multicultural reference data [38].

#### Parental PTSS

The Impact of Event Scale (IES) [39, 40] was used to assess parental PTSS within the first month postburn and 12 months postburn. This questionnaire assesses two dimensions of traumatic stress, namely symptoms of intrusion and avoidance. Both parents completed the Dutch version of the IES at both time points [41]. The IES consists of 15 items. Parents were asked to rate the frequency of symptoms they had experienced specifically in relation to their child’s burn event on a 4-point Likert scale (0-1-3-5). The total possible score ranged from 0 to 75, with higher scores representing higher levels of stress. Scores higher than 26 on the total scale were referred to as ‘clinically significant stress.’ In our sample, Cronbach’s alpha for the total IES was .86 (T1) and .87 (T2) for mothers and .86 (T1) and .90 (T2) for fathers.

#### Child and burn characteristics

Characteristics of the child (i.e., gender and age) and the burn (i.e., percentage TBSA, number of surgeries during initial hospitalization and length of stay in the hospital) were reported from the medical file. TBSA is the estimated percentage body surface area affected by second- and third-degree burns. The number of surgeries reflects the amount of skin grafting procedures the child has undergone. Parents provided information within the first month postburn on the place of the burn event (i.e., inside or outside the home) and the cause of the burn (e.g., hot fluid, flame, or contact with hot object).

### Statistical analyses

First, data were inspected for typing errors and missing values. Next, normality of the data was examined, and means, standard deviations, and intercorrelations among variables were calculated.

Second, prevalence of clinically significant parental PTSS was described and paired sample *t* tests were used to compare parental PTSS at T1 and T2. To answer our first hypothesis, pre- and postburn behavioral problems were compared to reference data [35, 38], with one-sample *t* tests.

Third, paths models were used to estimate direct and indirect associations among the constructs (Fig. 1) within a structural equation modeling framework, using Mplus 6.11 [42]. Four separate structural models were examined, because the relatively small sample size did not permit including all variables in a single model. The first two models included mother and child reports of internalizing and externalizing problems, respectively. Likewise, two separate models were fitted for father and child reports of internalizing and externalizing problems. Direct effects of preburn behavioral problems and parental PTSS within the first month postburn on postburn behavioral problems were modeled. The indirect (mediational) effect of parental stress within the first month postburn via parental stress symptoms 12 months postburn was examined: the total effect of the independent variable PTSS within the first month postburn, on the dependent variable behavioral problems, was subdivided into its direct effect on the dependent variable and its indirect effect on the dependent variable through the proposed mediator PTSS 12 months postburn. Covariates included child age, gender, and percentage TBSA. Postburn parent and child reports of behavioral problems were allowed to correlate. Because the sample size was too small for the relatively large amount of parameters, model constraints had to be imposed. Based on previous research and after checking the correlation matrix, specific (nonsensical) correlations between independent variables were constrained to zero. These included the correlations between parental PTSS at T1 and T2 and preburn behavioral problems, child age and preburn behavioral problems, child age and percentage TBSA, and preburn behavioral problems and percentage TBSA. All continuous independent variables (i.e., child age, percentage TBSA, preburn behavioral problems, and parental PTSS) were centered using the grand mean, and the dichotomous predictor child gender was labelled 0–1 (0 = boy).

As recommended by Preacher and Hayes [43], we employed a bootstrapping method (with *n* = 5000 bootstrap resamples) to assess the indirect effects and to control for nonnormally distributed data. Results of the path model were displayed using bootstrap bias-corrected 95 % confidence intervals (CI). Point estimates of effects were considered significant in case zero was not contained in the confidence interval. Relationships were tested two sided.

Missing data at T2 were estimated using full-information maximum likelihood (FIML). With this default method in Mplus, all available information is used to estimate missing data. As a consequence, we were able to include data from the total sample of 90 families. We assessed model fit with the *χ*^2^ statistic, the comparative fit index (CFI), the Tucker Lewis Index (TLI), and the root-mean-square of approximation (RMSEA). CFIs and TLIs above .90 [44] and RMSEAs less than .08 indicate a good fit and were used as the criteria for evaluating model fit beyond the *χ*^2^ statistic.

## Results

### Descriptive statistics and correlations

Table 2 shows the means, standard deviations, and intercorrelations of the variables. All variables were approximately normally distributed (skewness range −.20 to 1.57; kurtosis range −1.03 to 2.28), with the exception of percentage TBSA, which was positively skewed (skewness: 3.20) and had a high peak (kurtosis: 11.31).

**Table 2:** Intercorrelations and descriptive statistics (*n* = 90)

	1	2	3	4	5	6	7	8	9	10	11	12	13	14
1. Preburn behavioral problems M	–													
2. Preburn behavioral problems F	.55**	–												
3. PTSS 1 month postburn M	.11	.07	–											
4. PTSS 1 month postburn F	−.07	−.08	.20	–										
5. PTSS 12 months postburn M	.08	.14	.70**	.40*	–									
6. PTSS 12 months postburn F	−.02	.16	.47**	.44**	.52**	–								
7. Externalizing problems M	.50**	.39*	−.13	−.14	.10	.13	–							
8. Externalizing problems F	.70**	.61**	−.14	−.28	.09	.12	.75**	–						
9. Externalizing problems C	.24	.03	−.16	−.13	−.05	.11	.62**	.63**	–					
10. Internalizing problems M	.30*	.28	.28*	.13	.45**	.30	.30*	.23	−.07	–				
11. Internalizing problems F	.40*	.59**	.11	−.27	.35*	.26	.42*	.53**	.26	.65**	–			
12. Internalizing problems C	.09	−.15	.07	.13	.12	.15	.21	.11	.44**	.35*	.47*	–		
13. Child age	−.09	−.01	−.24*	−.12	−.23	−.15	−.09	−.03	−.15	−.13	−.15	−.19	–	
14. Percentage TBSA	.09	−.16	.17	.13	.09	−.09	−.19	−.18	−.27	−.05	−.27	−.26	.12	–
*M*	9.31	9.00	25.48	15.85	16.25	8.66	49.58	49.03	47.22	50.53	47.34	47.63	13.88	9.95
SD	5.94	6.04	14.34	12.45	12.20	10.53	10.18	10.03	10.13	8.92	9.72	11.03	2.43	12.26

*M* mother report, *F* father report, *C* child report, *PTSS* Posttraumatic stress symptoms, *TBSA* Total burned surface area

* *p* < .05, ** *p* < .01

The correlation between maternal and paternal reports of child behavioral problems at T1 was .55. For internalizing and externalizing problems at T2, these correlations were .65 and .75, respectively. These large correlations indicate that parental agreement on child behavior problems was high. Correlations between child-reported and mother-reported internalizing and externalizing problems were .35 and .62, respectively. For child–father agreement, these correlations were .47 (internalizing problems) and .63 (externalizing problems).

### Prevalence of parental PTSS and child behavioral problems

In the first month postburn, 47 % of the mothers reported clinically significant PTSS in relation to their child’s burn event. For fathers, this was 27 %. One year postburn, 27 % of the mothers and 8 % of the fathers reported clinically significant PTSS. For mothers and fathers, the reduction in stress symptoms from T1 to T2 was significant: *M*_mothersT1_ = 25.15, SD = 14.78, *M*_mothersT2_ = 16.25, SD = 12.20; *t* (54) = 6.18, *p* < .001; *M*_fathersT1_ (T1) = 15.00, SD = 12.90, *M*_fathersT2_ = 8.75, SD = 10.81; *t* (35) = 2.97, *p* = .005.

Mothers and fathers reported total preburn behavioral problems on the SDQ in the (sub)clinical range in 27 and 25 % of the children, respectively. Regarding postburn behavioral problems, CBCL reports of mothers and fathers indicated that, respectively, 16 and 17 % of children had externalizing problems in the (sub)clinical range. For internalizing problems, these percentages were 15–6 %. Finally, 12 % of children themselves reported postburn externalizing problems in the (sub)clinical range and 16 % reported internalizing problems in this range. The levels of agreement for the presence or absence of internalizing problems in the (sub)clinical range were 87 % for mothers and children, 90 % for fathers and children, and 84 % for mothers and fathers. For externalizing problems, these percentages were 87, 83, and 84 %, respectively.

Compared to reference data, preburn behavioral problems in our sample were not significantly different (mother reports: *p* = .11, Cohen’s *d* = .18; father reports: *p* = .19, Cohen’s *d* = .17). Regarding postburn behavioral problems, using the CBCL and YSR, in general, no significant differences between our sample and reference data were found and, on average, effect sizes indicated only small differences (internalizing problems, mother reports: *p* = .13, Cohen’s *d* = −.20, child reports: *p* = .09, Cohen’s *d* = −.27; externalizing problems, mother reports: *p* = .73, Cohen’s *d* = .05, father reports: *p* = .73, Cohen’s *d* = −.06 child reports: *p* = .27 Cohen’s *d* = −.16). One medium difference (*d* = −.40) was found for fathers, who reported significantly less internalizing problems in their child compared to parents from reference data: *M*_reference_ = 6.95, SD = 5.80, *M*_fathersT2_ = 4.82, SD = 4.83; *t* (32) = −2.54, *p* = .02.

### Path analyses

All four models showed adequate fit to the data, which is shown in Table 3. The results of the four path analyses are presented in Tables 4 and 5. Maternal reports of preburn behavioral problems were positively associated with their own reports of child internalizing problems 12 months postburn, although the association was at the borderline of significance (Table 4). For fathers, the association was significant (Table 5). In contrast, self-reported postburn internalizing problems by the child were not associated with preburn behavioral problems reported by mothers or fathers. This same pattern of results was obtained for externalizing problems.

**Table 3:** Model fit information for four path models

		CFI	TLI	RMSEA	*χ* ^2^	*df*	*p*
Model 1	Mother- and child reports of internalizing problems	.98	.92	.06	6.31	5	.28
Model 2	Mother- and child reports of externalizing problems	.98	.94	.05	6.25	5	.28
Model 3	Father- and child reports of internalizing problems	1.00	.99	.02	5.11	5	.40
Model 4	Father- and child reports of externalizing problems	1.00	1.00	.01	5.05	5	.41

**Table 4:** Relationship of preburn behavioral problems, maternal posttraumatic stress symptoms (PTSS), and covariates with mother- and child-reported behavioral problems 12 months postburn

	Model 1 Internalizing problems								Model 2 Externalizing problems							
	Mother reports				Child reports				Mother reports				Child reports			
	*B*	CI	SE	β	*B*	CI	SE	β	*B*	CI	SE	β	*B*	CI	SE	β
Direct effects																
Preburn behavioral problems	**0.50**	**0.00, 1.04** ^**a**^	.27	**.34**	0.27	−0.46, 1.05	.38	.15	**1.02**	**0.56, 1.48**	.24	**.57**	0.48	−0.20, 1.17	.35	.27
PTSS 1 month postburn	0.02	−0.20, 0.19	.10	.03	−0.06	−0.34, 0.25	.15	−.07	−0.16	−0.41, 0.04	.11	−.22	−0.15	−0.42, 0.17	.15	−.21
PTSS 12 months postburn	**0.29**	**0.06, 0.53**	.12	**.39**	0.14	−0.32, 0.57	.22	.15	0.23	−0.04, 0.52	.14	.26	0.13	−0.26, 0.50	.19	.15
Percentage TBSA	−0.07	−0.22, 0.04	.07	−.10	−**0.19**	−**0.50,** −**0.04**	.14	−**.22**	−**0.20**	−**0.33,** −**0.06**	.07	−**.24**	−**0.21**	−**0.43,** −**0.07**	.10	−**.26**
Child gender^b^	1.81	−5.43, 7.31	3.13	.09	3.25	−5.96, 12.55	4.74	.13	−3.42	−9.35, 2.23	2.97	−.14	−4.88	−11.44, 1.96	3.35	−.21
Child age	0.00	−0.91, 1.12	.51	.00	−0.67	−2.48, 1.06	.92	−.15	−0.64	−1.59, 0.29	.48	−.14	−0.98	−2.28, 0.31	.67	−.23
PTSS T1 →PTSS T2	**0.54**	**0.36, 0.68**	.08	**.64**	**0.54**	**0.36, 0.68**	.08	**.64**	**0.53**	**0.36, 0.68**	.08	**.64**	**0.53**	**0.36, 0.68**	.08	**.64**
Indirect effects																
PTSS T1 →PTSS T2 → behavioral problems	**0.16**	**0.04, 0.32**	.07	**.25**	0.07	−0.16, 0.32	.12	.10	0.12	−0.02, 0.30	.08	.16	0.07	−0.14, 0.27	.10	.10
Explained variance	30 %				13 %				44 %				24 %			

*CI* Bootstrap bias-corrected two-sided 95 % confidence interval. Effects that are statistically significant are written in bold, *TBSA* Total burned surface area

^a^ *p* = .06

^b^0 = boy, 1 = girl

**Table 5:** Relationship of preburn behavioral problems, paternal PTSS, and covariates with father- and child-reported behavioral problems 12 months postburn

	Model 3 Internalizing problems								Model 4 Externalizing problems							
	Father reports				Child reports				Father reports				Child reports			
	*B*	CI	SE	β	*B*	CI	SE	β	*B*	CI	SE	β	*B*	CI	SE	β
Direct effects																
Preburn behavioral problems	**0.79**	**0.21, 1.26**	.27	**.50**	−0.30	−1.05, .56	.42	−.17	**0.84**	**0.08, 1.37**	.32	**.53**	0.02	−0.80, .90	.44	.01
PTSS 1 month postburn	−**0.38**	−**0.79,** −**0.12**	.16	−**.50**	−0.05	−0.66, 0.41	.27	−.06	−0.23	−0.70, 0.10	.20	−.30	0.00	−0.55, 0.39	.23	−.01
PTSS 12 months postburn	**0.31**	**0.03, 0.68**	.16	**.35**	0.18	−0.52, 0.69	.31	.18	0.16	−0.27, 0.43	.16	.18	0.16	−0.44, 0.60	.25	.17
Percentage TBSA	0.07	−0.31, 0.40	.19	.09	−0.15	−0.73, 0.33	.24	−.16	0.03	−0.37, 0.66	.24	.03	−0.20	−0.65, 0.54	.28	−.23
Child gender^a^	6.45	−0.11, 11.95	3.09	.31	5.22	−5.30, 15.21	5.27	.23	0.81	−9.11, 8.07	4.25	.04	−4.83	−15.13, 3.34	4.62	−.22
Child age	−0.61	−2.04, 0.60	.66	−.15	0.75	−1.60, 2.64	1.06	.17	−0.52	−2.00, 0.98	.77	−.13	0.13	−1.89, 1.94	.95	.03
PTSS T1 →PTSS T2	**0.41**	**0.16, 0.75**	.15	**.47**	**0.41**	**0.16, 0.75**	.15	**.47**	**0.40**	**0.16, 0.76**	.15	**.47**	**0.40**	**0.16, 0.76**	.15	**.47**
Indirect effects																
PTSS T1 →PTSS T2 → behavioral problems	**0.13**	**0.02, 0.43**	.09	**.16**	0.07	−0.19, 0.39	.14	.08	.06	−0.07, 0.26	.08	.08	0.06	−0.15, 0.33	.12	.08
Explained variance	52 %				14 %				36 %				16 %			

*CI* Bootstrap bias-corrected two-sided 95 % confidence interval. Effects that are statistically significant are written in bold, *TBSA* Total burned surface area

^a^0 = boy, 1 = girl

Appraisals of PTSS within the first month postburn were associated with appraisals of PTSS 12 months postburn for mothers as well as fathers. Maternal reports of their PTSS within the first month postburn were not directly associated with their reports and child reports of internalizing and externalizing problems. However, maternal PTSS 12 months postburn had a significant positive relationship with their own reports of child internalizing problems. Paternal reports of their own PTSS within the first month postburn were associated with reporting less internalizing problems in their child. In contrast, their PTSS 12 months postburn had a positive relationship with their reports of internalizing problems. There were no significant direct relationships between mothers’ or fathers’ PTSS 12 months postburn and parental reports of child externalizing problems and child reports of internalizing and externalizing problems. In both parents, PTSS 12 months postburn significantly mediated the effect of PTSS within the first month postburn on their reports of child internalizing problems. No significant indirect effects were found for externalizing problems. In addition, no significant indirect effect of maternal or paternal PTSS within the first month postburn via PTSS 12 months postburn on child reports of internalizing and externalizing problems was found.

Child age and gender were not significant predictors of postburn behavioral problems. Moreover, no significant effects were found for the percentage TBSA in the models using father and child reports of internalizing and externalizing problems. However, a lower percentage of TBSA was associated with more externalizing problems reported by the mother, as well as with child self-reported internalizing and externalizing problems (in models 1 and 2). As this finding was unexpected, data were inspected to explain the relationships found. Two children with high percentages TBSA were identified (e.g., 59 and 72 % TBSA; two outliers which were found earlier) that had low to moderate ratings on all measures of behavioral problems. To check if these outliers possibly influenced the results, analyses for models 1 and 2 were repeated without the two outliers (results are not displayed, but are available upon request), which resulted in nonsignificant parameters in two of the three relationships. Therefore, the relationships found seemed to be especially due to the influence of two children with high percentages TBSA and low ratings of behavioral problems.

Preburn behavioral problems, parental PTSS 1 and 12 months postburn, percentage TBSA, child gender, and child age explained 13 to 52 % of variance in the four path models. Percentages were higher for parent reported problems, compared to child reports, which can be seen in Tables 4 and 5.

## Discussion

This study included child, mother, and father reports of postburn behavioral problems, hereby providing an encompassing family perspective on postburn adjustment. Results showed that overall, pre- and postburn behavioral problems were within normal limits. Depending on the informant, rates of (sub)clinically significant postburn internalizing and externalizing problems ranged from 6 to 17 %. Parents and children displayed rather similar perspectives on the presence or absence of (sub)clinical behavioral problems, with mother–child agreement in 87 % of the cases for internalizing and externalizing problems and father–child agreement in, respectively, 90 and 83 % of the cases. Findings further showed that postburn internalizing and externalizing problems were related to preburn behavioral functioning, but only if parents reported on postburn behavioral problems. Additionally, higher parental stress symptoms 12 months postburn were associated with more child internalizing problems as reported by mothers and fathers, but not as reported by children. In both parents, an indirect relationship was found from PTSS within the first month postburn via PTSS 12 months postburn to reporting internalizing problems in their child. Results of this study emphasize the use of a family systems perspective in research and clinical practice regarding pediatric burns.

As hypothesized using results of prior studies [6, 7], children and adolescents in our sample were not different from reference samples with regard to preburn behavioral problems and postburn internalizing and externalizing problems. As we only examined the long-term outcome, we cannot rule out the possibility that children and adolescents experienced transient internalizing and externalizing problems during or shortly after the period of hospitalization. Previous research has reported elevated symptoms during these periods [3–5]. Furthermore, we did not include measures of child acute and posttraumatic stress symptoms, while it has been shown before that a considerable part of children and adolescents experience these symptoms after burn injury [45, 46]. However, overall, the results of this study indicate that, on average, children and adolescents are not at risk to experience elevated levels of internalizing and externalizing problems 12 months after burn injury, which is a positive finding.

The inclusion of appraisals of preburn functioning in this study provides additional information about the adjustment of children and adolescents after burn injury. As hypothesized, our results showed that preburn behavioral problems were predictive of postburn internalizing and externalizing problems, from the parents’ perspective. As proposed [17], pretrauma psychological problems may continue to place children at a disadvantage with regard to their ability to cope and recover from the stressful event, hereby maintaining or increasing psychological problems. However, the role of preburn adjustment was not unequivocal in this study, as it was not predictive of child reports of postburn behavioral problems. In our study, parents appraised the preburn adjustment of the child, while child reports could have provided different views. Future studies are needed to investigate whether a similar relationship would be found for child-reported pre- and postburn behavioral problems.

This study showed that burn severity was not related to postburn behavioral problems in the models using father and child reports. This concurs with previous child studies that found no relation between burn size and postburn behavioral problems [6, 7, 18]. However, in the current study, a larger percentage TBSA was related to less behavioral problems in the models using mother and child reports. The counterintuitive finding was primarily due to two children with high percentages TBSA and low scores on behavioral problems. Percentage TBSA is only one aspect of burn severity; previous studies have shown a negative impact of visible scarring [7] or poor hand functioning [47] on postburn outcomes. However, in general, the results of the present study support the idea that larger burn size does not necessarily implicate a higher risk of postburn adjustment problems.

Conform our hypothesis, we found significant associations between parental PTSS 12 months postburn and parental reports of internalizing problems in their child. Different mechanisms may explain this relationship. A first possibility is that parents’ own levels of stress diminish their capacity to be responsive to the child’s needs, which may have an adverse impact on the child [19]. However, as these findings not held for child self-reports, it may be more likely that parental mental health affects the way in which parents interpret the behavior of their offspring. Previous studies have indicated that parental acute and posttraumatic stress reactions influence their assessment of child symptoms [28–30, 48]. Parents with higher PTSS might be more prone to perceive internalizing problems in their child, because they may have difficulty in differentiating their child’s reactions from their own [29]. As PTSS fall more within the spectrum of internalizing syndromes, this idea is supported by not finding significant associations between parental PTSS and reports of child externalizing problems in this study. The presence of an association between parental PTSS and reports of internalizing problems, but not externalizing problems, might also be due to the child’s internalizing problems being more difficult to observe [49] and therefore more prone to observational bias resulting from parental PTSS.

Although parental posttraumatic stress within the first month postburn (i.e., more acute stress) was not directly related to parent reports of child behavioral problems at 12 months, there was an indirect relationship of early PTSS via PTSS 12 months postburn to parental reports of child internalizing problems measured at 12 months. In concordance with previous studies, levels of parental PTSS at 1 and 12 months postburn were significantly related and suggest the possibility of chronification of PTSS [21]. The observed indirect nature of the relationship between early PTSS and parental reports of internalizing problems might indicate that only parents with chronic PTSS (in contrast to transient PTSS) are more prone to perceive higher internalizing problems in their child. Still, other studies found parental acute stress symptoms to be directly related to reports of young children’s 12 months postburn behavioral problems [10] and PTSS of school-aged children after a medical traumatic event [21]. Beyond the aforementioned relationships, in the present study, a negative association was found between paternal initial PTSS and fathers’ reports of child postburn internalizing problems. As no previous study has reported this finding, an explanation for this relationship is highly speculative. In spite of this unexpected result, the indirect effect found in this study suggests that parents’ higher acute stress symptoms that are not transient may be regarded a risk factor for long-term PTSS and consequent higher ratings of child postburn internalizing problems.

This multicenter study has a number of strengths, including the prospective design, the inclusion of fathers, the assessment of preburn functioning, and the relatively large sample size compared to other child burn studies. However, several limitations need to be considered too. First, we could only use information from two time points. Therefore, conclusions about directionality of effects cannot be drawn. For example, it is possible that children’s postburn internalizing problems contribute to parental PTSS, as parents struggle to deal with their protective feelings and guilt [50]. However, as we found no significant relationships with child reports of internalizing problems, this explanation appeared less likely. Second, it could be argued that parental PTSS may be a result of burn injury in parents themselves. However, as the incidence of injury in other family members (not specifying which family member was injured) was only 8 % in the present sample, this could not explain the high prevalence of parental PTSS (i.e., 47 % in mothers and 27 % in fathers within the first month postburn). We therefore assume that parental traumatic stress symptoms are mainly the consequence of perceived threat to the child’s life, witnessing the burn event or parental emotions related to appraisals about the trauma [25]. Last, as in most burn studies, the absolute sample size in this study was small, which may limit generalizability of the results. Future research is warranted to replicate the findings. As a consequence of the small sample size, there was also limited statistical power to investigate more parameters in this study and to investigate the maternal, paternal, and child reports in a single model. Attempts were made to include all parameters in a single model, but model fit was inappropriate. However, in the model concerned, nonsignificant equality constraints between fathers and mothers indicated no overall differences.

To increase the generalizability of our findings, future research is needed, especially regarding the relative contribution of preburn functioning to postburn outcomes as this was a vulnerability factor in this study. Further studies could also include pretrauma measures of resilience, such as prosocial behavior, personality characteristics, and positive family functioning. Furthermore, future studies are warranted to examine the mechanism explaining the relationship between parental PTSS and child postburn outcomes.

Results from this study may have implications for clinical burn practice. First, appraisals of preburn behavior shortly after the burn injury appear to be predictive of postburn problems from a parental perspective. This suggests that high-risk children may be identified in an early phase by the use of screening and that these children and their families may be followed up thereafter. However, the lack of evidence for the role of preburn behavior as perceived by parents, as well as the small total amount of variance explained in child self-reports of postburn behavioral problems, suggests that more insight is needed into determinants of the childs’ own appraisal of postburn problems. Aspects such as social support [51], coping style, and personality [52] may be important in this respect. Second, this study points to the role of chronic parental PTSS. Although parental acute stress symptoms in general are high and symptoms may largely decrease with time [25], a substantial amount of parents continue to experience clinically significant stress symptoms, as was shown again in the present study. Monitoring these symptoms in the aftermath of a pediatric burn event seems important for parental well-being but also for its impact on (the perception of) child postburn behavioral problems. Last, this study points to the role that choice of informants plays in assessing postburn adjustment. In the assessment of behavioral problems, no one’s informant report can be used as a ‘gold standard’ [53]. This implicates that, when possible, reports of multiple informants should be included, as they all provide valuable information. Possible discrepancies should be discussed and incorporated in determining treatment goals and decisions.

Overall, the results of this study suggest that child behavioral problems 12 months postburn as perceived by parents are associated with parental appraisals of child preburn problems as well as parental chronic PTSS. Therefore, we recommend clinical burn practice to use a family perspective, by specifically including parents in assessment and intervention.

## References

[1] Pope S, Solomons W, Done D, Cohn N, Possamai A (2007). Body image, mood and quality of life in young burn survivors. Burns.

[2] Lawrence JW, Rosenberg L, Mason S, Fauerbach JA (2011). Comparing parent and child perceptions of stigmatizing behavior experienced by children with burn scars. Body Image.

[3] Delgado Pardo G, Garcia IM, Marrero FR, Gomez-Cia T (2008). Psychological impact of burns on children treated in a severe burns unit. Burns.

[4] Liber JM, List D, Van Loey NE, Kef S (2006). Internalizing problem behavior and family environment of children with burns: a Dutch pilot study. Burns.

[5] Delgado Pardo G, Garcia IM, Gomez-Cia T (2010). Psychological effects observed in child burn patients during the acute phase of hospitalization and comparison with pediatric patients awaiting surgery. J Burn Care Res.

[6] Blakeney P, Meyer W, Moore P, Broemeling L, Hunt R, Robson M, Herndon D (1993). Social competence and behavioral problems of pediatric survivors of burns. J Burn Care Rehabil.

[7] Willebrand M, Sveen J, Ramklint MD, Bergquist RN, Huss MD, Sjoberg MD (2011). Psychological problems in children with burns—parents’ reports on the Strengths and Difficulties Questionnaire. Burns.

[8] Landolt MA, Grubenmann S, Meuli M (2000). Psychological long-term adjustment in children with head burns. J Trauma.

[9] Rosenberg L, Blakeney P, Thomas CR, Holzer CE, Robert RS, Meyer WJ (2007). The importance of family environment for young adults burned during childhood. Burns.

[10] Bakker A, van der Heijden PG, van Son MJ, van de Schoot R, Vandermeulen E, Helsen A, Van Loey NE (2014). The relationship between behavioural problems in preschool children and parental distress after a paediatric burn event. Eur Child Adolesc Psychiatry.

[11] Graf A, Schiestl C, Landolt MA (2011). Posttraumatic stress and behavior problems in infants and toddlers with burns. J Pediatr Psychol.

[12] Kent L, King H, Cochrane R (2000). Maternal and child psychological sequelae in paediatric burn injuries. Burns.

[13] McKibben JB, Bresnick MG, Askay SAW, Fauerbach JA (2008). Acute stress disorder and posttraumatic stress disorder: a prospective study of prevalence, course, and predictors in a sample with major burn injuries. J Burn Care Res.

[14] Van Loey NE, Oggel A, Goemanne A-S, Braem L, Vanbrabant L, Geenen R (2014). Cognitive emotion regulation strategies and neuroticism in relation to depressive symptoms following burn injury: a longitudinal study with a 2-year follow-up. J Behav Med.

[15] Öster C, Sveen J (2014). The psychiatric sequelae of burn injury. Gen Hosp Psychiatry.

[16] Meyer WJ, Robert R, Murphy L, Blakeney PE (2000). Evaluating the psychosocial adjustment of 2-and 3-year-old pediatric burn survivors. J Burn Care Res.

[17] Cox CM, Kenardy JA, Hendrikz JK (2008). A meta-analysis of risk factors that predict psychopathology following accidental trauma. J Spec Pediatr Nurs.

[18] Landolt MA, Grubenmann S, Meuli M (2002). Family impact greatest: Predictors of quality of life and psychological adjustment in pediatric burn survivors. J Trauma.

[19] Scheeringa MS, Zeanah CH (2001). A relational perspective on PTSD in early childhood. J Trauma Stress.

[20] De Young AC, Kenardy JA, Cobham VE (2011). Trauma in early childhood: a neglected population. Clin Child Fam Psychol Rev.

[21] Landolt MA, Ystrom E, Sennhauser FH, Gnehm HE, Vollrath ME (2012). The mutual prospective influence of child and parental post-traumatic stress symptoms in pediatric patients. J Child Psychol Psychiatry.

[22] Alisic E, Jongmans MJ, van Wesel F, Kleber RJ (2011). Building child trauma theory from longitudinal studies: a meta-analysis. Clin Psychol Rev.

[23] Ostrowski SA, Christopher NC, Delahanty DL (2007). Brief report: the impact of maternal posttraumatic stress disorder symptoms and child gender on risk for persistent posttraumatic stress disorder symptoms in child trauma victims. J Pediatr Psychol.

[24] McFarlane AC (1987). Posttraumatic phenomena in a longitudinal study of children following a natural disaster. J Am Acad Child Adolesc Psychiatry.

[25] Bakker A, Van der Heijden PG, Van Son MJ, Van Loey NE (2013). Course of traumatic stress reactions in couples after a burn event to their young child. Health Psychol.

[26] De Young AC, Hendrikz J, Kenardy JA, Cobham VE, Kimble RM (2014). Prospective evaluation of parent distress following pediatric burns and identification of risk factors for young child and parent posttraumatic stress disorder. J Child Adolesc Psychopharmacol.

[27] McGarry S, Girdler S, McDonald A, Valentine J, Wood F, Elliott C (2013). Paediatric medical trauma: the impact on parents of burn survivors. Burns.

[28] Smith P, Perrin S, Yule W, Rabe-Hesketh S (2001). War exposure and maternal reactions in the psychological adjustment of children from Bosnia-Hercegovina. J Child Psychol Psychiatry.

[29] Kassam-Adams N, Garcia-Espana JF, Miller VA, Winston F (2006). Parent-child agreement regarding children’s acute stress: the role of parent acute stress reactions. J Am Acad Child Adolesc Psychiatry.

[30] Pan R, Egberts MR, Nascimento LC, Rossi LA, Vandermeulen E, Geenen R, Van Loey NE (2015). Health-related quality of life in adolescent survivors of burns: agreement on self-reported and mothers’ and fathers’ perspectives. Burns.

[31] Achenbach TM (1991). Manual for the Youth Self-Report and 1991 profile.

[32] Goodman R (1997). The Strengths and Difficulties Questionnaire: a research note. J Child Psychol Psychiatry.

[33] Goodman R, Ford T, Simmons H, Gatward R, Meltzer H (2000). Using the Strengths and Difficulties Questionnaire (SDQ) to screen for child psychiatric disorders in a community sample. Br J Psychiatry.

[34] Goodman R (2001). Psychometric properties of the Strengths and Difficulties Questionnaire. J Am Acad Child Adolesc Psychiatry.

[35] Meltzer H, Gatward R, Goodman R, Ford T (2000). Mental health of children and adolescents in Great Britain.

[36] Achenbach TM (1991). Manual for the Child Behavior Checklist/4-18 and 1991 profiles.

[37] Verhulst FC, van der Ende J (2013) Handleiding ASEBA. Vragenlijsten voor leeftijden 6 tot en met 18 jaar. ASEBA Nederland, Rotterdam

[38] Achenbach TM, Rescorla LA (2007). Multicultural supplement to the manual for the ASEBA school-age forms & profiles.

[39] Horowitz M, Wilner N, Alvarez W (1979). Impact of Event Scale: A measure of subjective stress. Psychosom Med.

[40] Sundin EC, Horowitz MJ (2002). Impact of Event Scale: psychometric properties. Br J Psychiatry.

[41] Brom D, Kleber RJ (1985). De Schok Verwerkings Lijst [The Impact of Event Scale]. Ned Tijdschr Psychol.

[42] Muthén LK, Muthén BO (2010). Mplus User‘s Guide.

[43] Preacher KJ, Hayes AF (2008). Asymptotic and resampling strategies for assessing and comparing indirect effects in multiple mediator models. Behav Res Methods.

[44] Kline RB (2011). Principles and practice of structural equation modeling.

[45] Saxe G, Stoddard F, Chawla N, Lopez CG, Hall E, Sheridan R, King D, King L (2005). Risk factors for acute stress disorder in children with burns. J Trauma Dissociation.

[46] Landolt MA, Buehlmann C, Maag T, Schiestl C (2009). Brief report: quality of life is impaired in pediatric burn survivors with posttraumatic stress disorder. J Pediatr Psychol.

[47] Baker CP, Russell WJ, Meyer W, Blakeney P (2007). Physical and psychologic rehabilitation outcomes for young adults burned as children. Arch Phys Med Rehabil.

[48] Shemesh E, Newcorn JH, Rockmore L, Shneider BL, Emre S, Gelb BD, Rapaport R, Noone SA, Annunziato R, Schmeidler J (2005). Comparison of parent and child reports of emotional trauma symptoms in pediatric outpatient settings. Pediatrics.

[49] Achenbach TM, McConaughy SH, Howell CT (1987). Child/adolescent behavioral and emotional problems: implications of cross-informant correlations for situational specificity. Psychol Bull.

[50] Scheeringa MS, Zeanah CH (2008). Reconsideration of harm’s way: onsets and comorbidity patterns of disorders in preschool children and their caregivers following Hurricane Katrina. J Clin Child Adolesc Psychol.

[51] Bamum D, Snyder C, Rapoff M, Mani M, Thompson R (1998). Hope and social support in the psychological adjustment of pediatric bum survivors and matched controls. Child Health Care.

[52] Liber JM, Faber AW, Treffers PDA, Van Loey NEE (2008). Coping style, personality and adolescent adjustment 10 years post-burn. Burns.

[53] De Los Reyes A, Kazdin AE (2005). Informant discrepancies in the assessment of childhood psychopathology: a critical review, theoretical framework, and recommendations for further study. Psychol Bull.

